# Evaluation of green tea extract as a safe personal hygiene against viral infections

**DOI:** 10.1186/s13036-017-0092-1

**Published:** 2018-01-08

**Authors:** Yun Ha Lee, Yo Han Jang, Young-Seok Kim, Jinku Kim, Baik Lin Seong

**Affiliations:** 1 Department of Biotechnology, College of Life Science and Biotechnology, Yonsei University, Seoul, South Korea; 2 Department of Biological and Chemical Engineering, College of Science and Technology, Hongik University, Sejong, South Korea; 3 Peachchem Co. Ltd., Sejong, South Korea; 4 Vaccine Translational Research Center, Yonsei University, Seoul, South Korea

**Keywords:** Green tea extract, Catechins, Influenza virus, Antiviral, Antioxidant

## Abstract

**Background:**

Viral infections often pose tremendous public health concerns as well as economic burdens. Despite the availability of vaccines or antiviral drugs, personal hygiene is considered as effective means as the first-hand measure against viral infections. The green tea catechins, in particular, epigallocatechin-3-gallate (EGCG), are known to exert potent antiviral activity. In this study, we evaluated the green tea extract as a safe personal hygiene against viral infections.

**Results:**

Using the influenza virus A/Puerto Rico/8/34 (H1N1) as a model, we examined the duration of the viral inactivating activity of green tea extract (GTE) under prolonged storage at various temperature conditions. Even after the storage for 56 days at different temperatures, 0.1% GTE completely inactivated 10^6^ PFU of the virus (6 log_10_ reduction), and 0.01% and 0.05% GTE resulted in 2 log_10_ reduction of the viral titers. When supplemented with 2% citric acid, 0.1% sodium benzoate, and 0.2% ascorbic acid as anti-oxidant, the inactivating activity of GTE was temporarily compromised during earlier times of storage. However, the antiviral activity of the GTE was steadily recovered up to similar levels with those of the same concentrations of GTE without the supplements, effectively prolonging the duration of the virucidal function over extended period. Cryo-EM and DLS analyses showed a slight increase in the overall size of virus particles by GTE treatment. The results suggest that the virucidal activity of GTE is mediated by oxidative crosslinking of catechins to the viral proteins and the change of physical properties of viral membranes.

**Conclusions:**

The durability of antiviral effects of GTE was examined as solution type and powder types over extended periods at various temperature conditions using human influenza A/H1N1 virus. GTE with supplements demonstrated potent viral inactivating activity, resulting in greater than 4 log_10_ reduction of viral titers even after storage for up to two months at a wide range of temperatures. These data suggest that GTE-based antiviral agents could be formulated as a safe and environmentally friendly personal hygiene against viral infections.

## Background

Virus infections continue to pose major public health concerns with the possibility of epidemics and pandemics [1]. A recent outbreak of Ebola virus in 2014 and the global circulation of the pandemic influenza of swine origin (pH1N1) in 2009 resulted in numerous deaths and hospitalizations [2, 3]. Middle East respiratory syndrome (MERS) transmitted from camels to humans, and the human to human infections are being amplified in nosocomial setting in addition to direct household or community-wide transmissions [4, 5]. In addition, although probably less deadlier than Ebola virus, a food-borne noroviral infection causes gastroenteritis in humans, claiming millions of illnesses and hospitalizations, with occasional mortalities [6]. Most of emerging viral diseases are zoonotic in nature, posing unmet needs for personal hygiene and disinfectants for both humans and livestock.

Control and preventive actions against viral infections in human comprises vaccination, antiviral treatment, and personal hygiene. While vaccination and antiviral drugs are often considered very effective for preventing and controlling viral infections, personal hygiene provides much cost-effective alternatives to preventing such infections [7, 8]. In general, personal hygiene usually involves hand washing with soap or a hand sanitizer, mostly alcohol-based, due to inhibitory activities of alcohol against viruses [9, 10]. However, due to rapid evaporation upon air exposure, a short incubation with alcohol (3–95%) was found ineffective in reducing the viral activity [11]. In addition, there was a concern of fire safety and toxicity for the use of high alcohol content in hand sanitizers [12]. Furthermore, the United States Center for Disease Control and Prevention (CDC) reported that alcohol-based hand sanitizer use was a risk factor in long-term care facilities for norovirus [13]. Meanwhile, alternative hand sanitizers based on quaternary ammonium compounds have been used, but their toxicity and environmental concerns have been raised for its use in personal hygiene [14, 15]. Therefore, effective and environmental friendly compounds for sanitizers have been sought for safe human use to prevent microbial infections [16, 17].

**Fig. 1:**
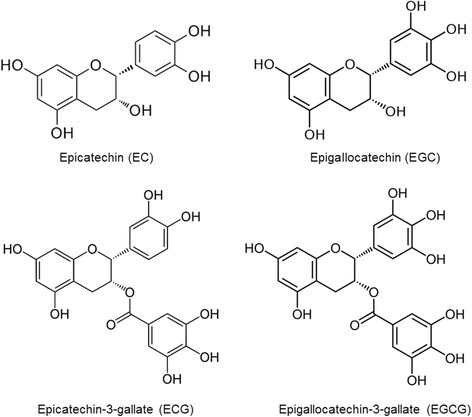
Chemical structures of green tea catechins. Chemical structures of epicatechin (EC), epigallocatechin (EGC), epicatechin-3-gallate (ECG), and epigallocatechin-3-gallate (EGCG) are shown

Green tea (*Camellia sinensis)* has been known to confer many benefits to human health [18–21]. The polyphenolic catechins in green tea are composed of epigallocatechin-3-gallate (EGCG), epigallocatechin (EGC), epicatechin-gallate (ECG), and epicatechin (EC) (Fig. 1), among which, EGCG is most abundant and exerts a wide range of physiological and pharmacological activities. Most prominently, EGCG exhibits antiviral activity in vitro against a variety of viruses of *Retroviridae*, *Orthomyxoviridae*, and *Flaviviridae,* including important human infectious pathogens such as human immunodeficiency virus (HIV), influenza A virus, and hepatitis C virus [22]. EGCG potently exerts inhibition of influenza virus replication [23, 24], interferes with HCV entry [25, 26], and inactivates Herpes simplex virus 1 (HSV-1) and HSV-2 at acidic and neutral pHs [27, 28]. In addition, EGCG was shown to block the enzymatic activity of the HIV-1 reverse transcriptase [29]. One of proposed mechanisms for the antiviral activity of EGCG is the covalent modification of proteins by EGCG by autoxidation process, in which EGCG is oxidized to form EGCG quinone by autoxidation, which in turn can react with the nucleophilic thiol group of a cysteine residue to form EGCG-protein complex [30]. Furthermore, it has been proposed that the autoxidation of catechins involves oxygen radical and molecular oxygen [31]. Although numerous previous studies have investigated antiviral activities of green tea extract or purified catechin components against viruses, there have been few reports with respect to developing disinfectants of public and personal hygiene. For practical use at home and in the field, a long-term stability at various working conditions is essential. Toward finding a safe and effective hygiene agent against viruses, we evaluated the durability of antiviral effects of green tea extract (GTE) as a powder type and a solution type over extended periods at various temperature conditions using human influenza A/H1N1 virus. The effect of antioxidant additive such as ascorbic acid further suggests that the virucidal function is mediated by the prooxidant activities of catechins.

## Methods

### Cell line and influenza virus

MDCK (Madin*-*Darby Canine kidney) were obtained from ATCC (American Type Culture Collection) and cultured in MEM (minimal essential medium, HyClone) supplemented with 10% FBS (fetal bovine serum, HyClone), at 37 °C in 5% CO_2_. Influenza virus A/Puerto Rico/8/34 (H1N1) (PR8) viruses were propagated in 11-day-old chicken embryos. The allantoic fluids were harvested and filtered by a syringe filter with a pore size of 0.2 μm and stored at a freezer (−80 °C) until use.

### Green tea extract

Green tea extract (GTE) was provided as powder form from Amore-pacific Co, Korea as described previously [32]. Briefly, green tea leaves were infused with 75 °C distilled water in the ratio of 1:7 (*w*/*w*). After 20 min of infusion, the tea extract was quickly separated from the tea leaves by filtration and the green tea extract (GTE) was freeze-dried. The composition was analyzed by C18 reverse phase column chromatography (elution with 22% THF at the flow of 1 ml/min). The GTE was composed of caffeine (5.48%), gallic acid (0.22%), GC (1.95%), EGC (10.22%), catechin (0.35%), EGCG (9.11%), EC (2.51%), and GCG (0.88%), as determined by HPLC. GTE solution was prepared by adding water to the powder and filtering it through a 0.2 μm syringe filter.

### Antiviral effect of GTE solution and powder

GTE powder was dissolved in distilled water to make 1% (10 mg/ml) solution and serially diluted to make 0.1%, 0.05%, 0.01%, and 0.001% solutions. The solutions were stored at various temperatures (4 °C, 25 °C, and 37 °C), and aliquots were taken at predetermined time-points (6 hours (h), 10 h, 1 day (d), 2 d, 4 d, 7 d, 14 d, 28 d, and 56 d) and immediately kept frozen at −20 °C until use. Equal volume of PR8 virus (10^6^ PFU dissolved in PBS) was added to the GTE solutions and incubated for six h at 25 °C before determining the viral titers. Residual viral titers were determined by plaque assay on MDCK cells at 37 °C, as described previously [33, 34]. MDCK cells in 12-well culture plates were infected with 200 μl of 10-fold serially diluted virus samples and incubated for 1 h on a shaker at room temperature (RT). The inoculums were removed and the cells were overlaid with medium containing 1% low-melting agarose, DMEM, and 10 μg/ml trypsin. The 12-well plates were incubated at 37 °C in 5% CO_2_ incubator. After three days of the incubation, the virus plaques were counted. The GTE power was stored at 25 °C and was taken at 1, 4, 8, and 16 weeks and kept at −20 °C until use. The GTE powders were dissolved in distilled water to make 1% (10 mg/ml) solution and diluted to 0.1% concentration. Then, equal volume of PR8 virus solution (10^6^ PFU/ml) was added, and the mixture was further incubated for six h at 25 °C. Residual viral titers were determined by plaque assay on MDCK cells at 37 °C.

### Antiviral effect and stability of GTE with supplements

GTE solutions (0.01, 0.05, and 0.1%) were supplemented with 2% citric acid, 0.1% sodium benzoate, and 0.2% ascorbic acid to make GTE-mix. The GTE-mix solutions were stored at 25 °C and 37 °C for up to 56 days. The GTE-mix taken at various time-points were incubated with 10^6^ PFU of PR8 virus at 25 °C for six h for viral inactivation. Residual viral titers were measured by plaque assay on MDCK cells. To determine the chemical stability of green tea catechins, 0.1% GTE and GTE-mix were stored at 25 °C for up to 56 days. Aliquots were taken at pre-determined time-points and were kept frozen at −20 °C until use. The concentrations of catechins at various time-points were analyzed by gas chromatography (GC) with a Hewlett–Packard (HP) column equipped with a FID detector, using helium as a carrier gas. Identification and quantification of the GC peaks was accomplished by GC/MS analysis with an Agilent HP 5973 GC/Mass Spectrometer (Agilent Technologies, USA).

### Analysis of the morphology of influenza virus by DLS and cryo-EM

The size distribution of influenza virus treated with GTE or PBS was measured by dynamic light scattering (DLS). 5 **×** 10^7^ PFU/mL of PR8 virus was treated with 0.1% GTE or PBS for 24 h at 25 °C. After the treatment, the virus samples were applied to the Particle size & Zeta potential Anlayzer (ELS-2000ZS, Otsuka Electronics, Japan). The size distribution was measured twice with accumulation time 200 in PBS solvent at 25 °C. The morphology of the virus treated with GTE was analyzed by cryo-electron microscopy (cryo-EM). Purified influenza viruses were loaded onto the Formvar Film 200 copper grid (Electron Microscopy Sciences). After 60 s of sample adsorption, the grid was negative stained with 1% uranyl acetate. Sample vitrification was performed using Vitrobot (FEI), and the vitrified sample was imaged using a CryoTecnai F20 transmission electron microscope (FEI).

## Results

### Viral inactivation activity of GTE against influenza virus

**Fig. 2:**
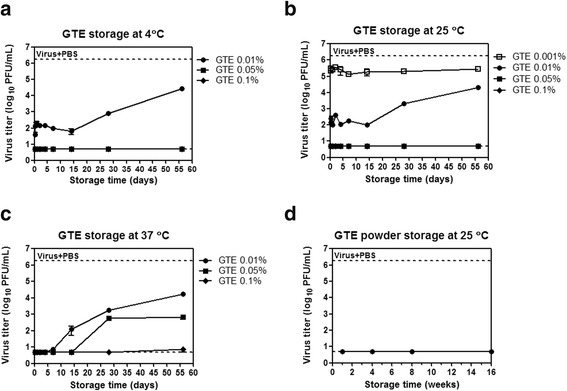
Maintenance of viral inactivation activity of GTE. **a**-**c** Maintenance of viral inactivation activity of GTE solution. 0.01, 0.05, and 0.1% GTE solutions were stored at 4 °C (**a**), 25 °C (**b**), and 37 °C (**c**), and 0.001% GTE was incubated at 25 °C. The GTE solutions incubated for different time points were mixed with 10^6^ PFU of PR8 virus and further incubated for six h at 25 °C for viral inactivation. Residual viral titers were determined by plaque assay on MDCK cells. **d** Maintenance of viral inactivation activity of GTE powder. GTE powder was stored for 1 − 16 weeks at 25 °C, and then dissolved in distilled water to make 0.1% GTE solutions. The GTE solutions were incubated with 10^6^ PFU of PR8 virus for six h at 25 °C for viral inactivation. Residual viral titers were determined by plaque assay on MDCK cells. As a control, the virus was incubated with PBS under the same conditions, and the viral titer was indicated by upper dashed lines. Data are the mean of two independent experiments. Detection limit of plaque assay is 5, and error bars indicate the standard deviation (SD)

First, we examined how long the viral inactivation activity of GTE was maintained after storage in different conditions. Various concentrations of GTE solutions were stored at 4 °C, 25 °C, and 37 °C for 6 h to 56 days, and the GTE solutions stored for different times were tested for their viral inactivating activities against an influenza virus A/Puerto Rico/8/34 (H1N1) (PR8). The results showed that 0.05% and 0.1% GTE solutions, stored at 4 °C and 25 °C as long as 56 days, maintained potent viral inactivating activities, completely removing the viral plaque-forming ability of 10^6^ PFU of the viruses (Fig. 2a & b). 0.01% GTE solutions stored for 14 days at 4 °C and 25 °C also demonstrated potent viral inactivating activity, resulting in approximately 4 log_10_ reduction of viral titers. 0.01% GTE solutions exhibited reduced viral inactivating activity following the storage more than 14 days at 4 °C and 25 °C, but still could inactivate the viruses (> 1 log_10_ reduction) even after 56 days of storage (Fig. 2a & b). 0.1% GTE solution stored at 37 °C also showed potent inactivating activity, although the complete removal of 10^6^ PFU viruses was not achieved when the GTE solution was stored for 56 days (Fig. 2c). 0.01% GTE solutions stored for less than 4 days and 0.05% GTE solutions stored for less than 14 days at 37 °C completely inactivated the viruses, but their activities decreased along storage time (Fig. 2c). The inactivating activity of 0.05% GTE solution was reduced by storage at 37 °C, as compared to those at 4 °C and 25 °C, in which complete inactivation was observed by the same concentration of GTE. 0.001% GTE stored at 25 °C demonstrated constant levels of weak inactivating activities (<1 log_10_ reduction), irrespective of storage times (Fig. 2b). The data showed that GTE was able to inactivate the influenza virus in a concentration-dependent manner. Of note, the potency of inactivating activity was gradually weakened following the storage more than 14 days, especially at lower concentrations of GTE (0.01% and 0.05%). However, ~2 log_10_ reduction of viral infectivity was still maintained over 56 days of incubation. Therefore, it can be concluded that the viral inactivating activity of GTE solution over 0.01% were robust and stable for at least two months as a solution type disinfectant even at hot weather conditions like in summer.

We further examined the maintenance of viral inactivating activity of GTE stored in dried powder. GTE powder was stored at 25 °C for up to 16 weeks, and the GTE powder taken at different time-points was dissolved in distilled water to make a 0.1% GTE solution. The 0.1% GTE solutions were incubated with 10^6^ PFU of PR8 virus at 25 °C for six h for viral inactivation. The results showed that, despite the storage for 16 weeks, GTE powder was able to inactivate the virus completely (Fig. 2d), indicating that the antiviral effects of GTE powder could be stably maintained over prolonged storage.

### Viral inactivating activity of GTE with supplements

**Fig. 3:**
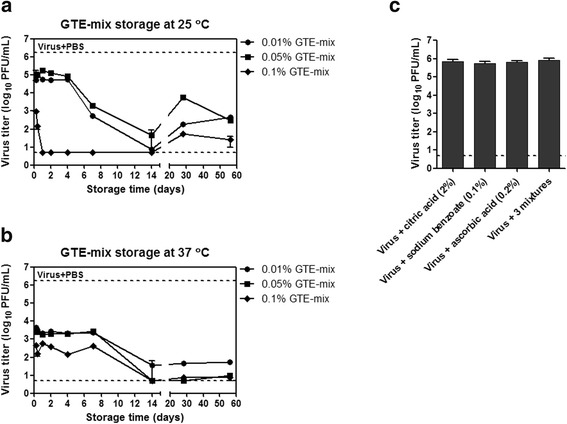
Maintenance of viral inactivation activity of GTE solution with supplements. 0.01, 0.05, and 0.1% GTE solutions were incubated at 25 °C (**a**) or 37 °C (**b**) in the presence of 2% citric acid, 0.1% sodium benzoate, and 0.2% ascorbic acid (GTE-mix). The GTE-mix solutions incubated for different times were mixed with 10^6^ PF of PR8 virus for six h at 25 °C for viral inactivation. Residual viral titers were determined by plaque assay on MDCK cells. As a control, the virus was incubated with PBS, and the viral titer was indicated by upper dashed lines. **c** As another controls, the virus was treated with the individuals of the supplement or with the mixture of three agents. Data are the mean of two independent experiments. Detection limit of plaque assay is 5, and error bars indicate the SD

Based on the previous report that ascorbic acid stabilized green tea catechins [35], we examined the effects of the addition of common food preservatives such as ascorbic acid, citric acid, and sodium benzoate on the viral inactivating activity of GTE. 0.01%, 0.05%, and 0.1% of GTE solutions were supplemented with 2% citric acid, 0.1% sodium benzoate, and 0.2% ascorbic acid (GTE-mix), and the mixtures were stored at 25 °C or 37 °C for six h–56 days. The GTE-mix solutions taken at various time-points were incubated with 10^6^ PFU of PR8 virus for six h at 25 °C for viral inactivation. The results show that, on a short-term period up to one week, the potency of the antiviral activity was compromised, but still enabled the reduction of the viral titers by 1–6log_10_ depending on GTE-mix concentrations. The reduction of the antiviral potency was more apparent at lower temperature of 25 °C (Fig. 3a), and at lower concentrations of GT-mix of 0.01% and 0.05% (Fig. 3a, b). The data also showed that, in the presence of an antioxidant such as ascorbic acid, the potency of GTE-mediated antiviral activity was significantly reduced. For example, the antiviral activities of 0.1% GTE-mix solutions stored at both temperatures were not as strong as 0.1% GTE that completely inactivated the virus (Fig. 2a–c). Likewise, 0.05% and 0.01% GTE-mix solutions also demonstrated considerably compromised antiviral activities, as compared to the same concentrations of GTE solutions (Fig. 2b & c). The reduced antiviral activities of GTE-mix solutions were more pronounced when stored for relatively short periods less than one week. Interestingly, however, the antiviral activities of 0.1% GTE-mix stored at 25 °C became complete after 1 day of storage, and 0.01% and 0.05% GTE-mix also steadily restored their antiviral activities at 14 days to the similar levels with the same concentrations of GTE (Figs. 2b and 3a). The similar phenomena were observed in the GTE-mix solutions stored at 37 °C. All the GTE-mix solutions stored for less than 14 days exhibited reduced antiviral activities but restored their activity to the similar levels to those of the same concentrations of GTE solutions at 14 days, and, notably, remained potent up to 56 days (Figs. 2c and 3b). Thus, storage time-dependent inactivating activities of GTE-mix solutions were in clear contrast to those of GTE solutions, where the initial antiviral strength was high for up to two weeks but exhausted over prolonged storage (Fig. 2). None of the individual supplements nor their mixture showed inactivating activity against the viruses (Fig. 3c), indicating that the inactivation was solely due to the GTE. The results suggest that antioxidants such as ascorbic acid, although compromising the initial antiviral strength, could be used for prolonging the shelf-life of GTE-based antiviral disinfectant.

### Effects of supplements on the kinetics of viral inactivation by GTE

**Fig. 4:**
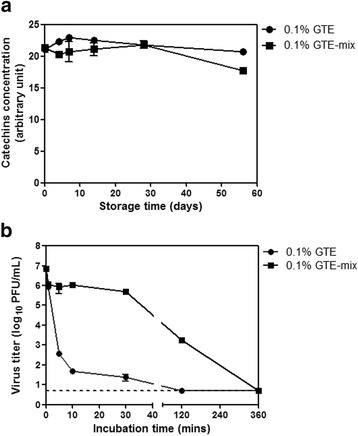
Effects of supplements on the kinetics of viral inactivation by GTE. **a** Chemical stability of green tea catechins. 0.1% GTE and GTE-mix solutions were stored at 25 °C for up to 56 days. The samples were obtained at various time-points and catechins concentration was measure by GC. **b** Kinetics of viral inactivation by GTE and GTE-mix. 0.1% GTE and GTE-mix solutions were incubated at for 24 h at 25 °C. After the incubation, the GTE and GTE-mix solutions were mixed with 10^6^ PFU of PR8 virus and further incubated at 25 °C for 0, 1, 5, 10, 30, 120, and 360 mins for viral inactivation. Residual viral titers at each time-point were determined by plaque assay on MDCK cells. Data are the mean of two independent experiments. Detection limit of plaque assay is 5, and error bars indicate the SD

We further examined whether the addition of supplements (citric acid, sodium benzoate, and ascorbic acid) influenced the chemical stability of green tea catechins that were known to exert the antiviral activity. GTE and GTE-mix solutions were stored at 25 °C for up to 56 days, and the concentrations of catechins in the solutions were determined by GC/MS analysis. The catechins were stably maintained regardless of the presence of the supplements (Fig. 4a), showing that the addition of the supplements did not affect the catechins stability in the GTE solution. Next, we monitored the kinetics of viral inactivation of GTE in the presence of the supplements. Based on the result in Fig. 3a, we further compared the inactivation kinetics between GTE and GTE-mix solutions. 0.1% of GTE and GTE-mix solutions were stored at 25 °C for 1 day, and the solutions were then further incubated with 10^6^ PFU of PR8 virus at 25 °C for 0–360 min for viral inactivation. The viral titers in the mixtures taken at different time-points were determined by plaque assay to observe the kinetics of time-dependent viral inactivation. Viral inactivation by GTE solution was rapid, resulting in greater than 4 log_10_ reduction of viral titers within five mins after the incubation, and a complete inactivation was achieved after 120 mins after the incubation (Fig. 4b). On the other hand, GTE-mix solution resulted in only 1 log_10_reduction of the viral titers within 30 mins, and complete inactivation was achieved after 360 mins of the incubation (Fig. 4b), clearly showing that supplements added to the GTE solution interfered with catechin-mediated viral inactivation. Taken together, the addition of the supplements to GTE solution resulted in the delay of the exhaustion of the inactivating activity of GTE, thus enabling a long-term maintenance of its antiviral function.

### Morphology of influenza virus treated with GTE

**Fig. 5:**
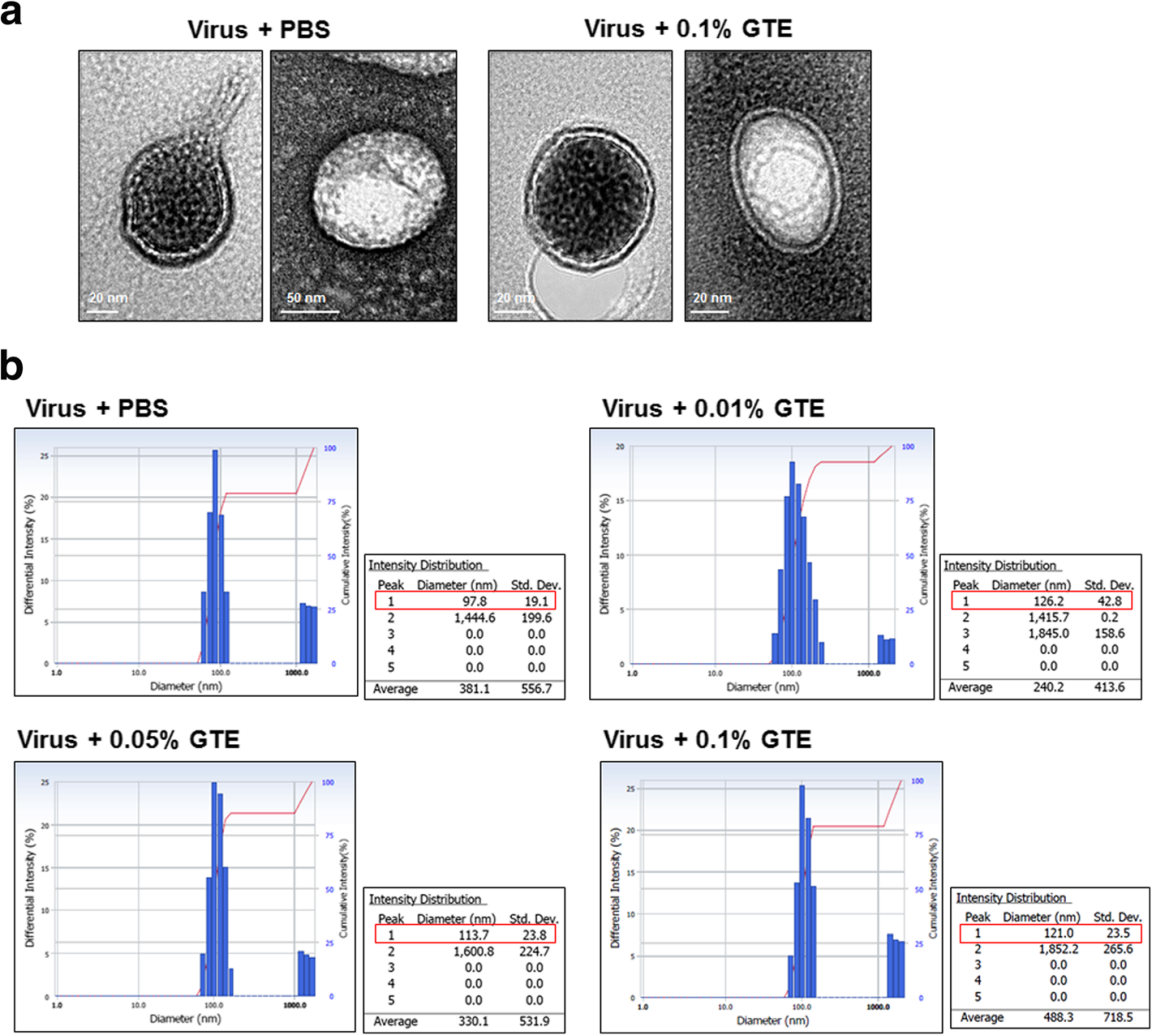
Morphology of influenza virus treated with GTE. **a** Cryo-EM images of PR8 virus treated with PBS or GTE. 5 **×** 10^7^ PFU of PR8 virus was incubated with PBS or 0.1% GTE 0.1% for 24 h at 25 °C before observation under cryo-EM. Two images of the virus treated either PBS or GTE 0.1% are shown. **b** Analysis of the size distribution of PR8 virus treated with GTE or PBS by dynamic light scattering (DLS). 5 **×** 10^7^ PFU of PR8 virus was incubated with PBS or GTE (0.01, 0.05, and 0.1%) for 24 h at 25 °C. After the incubation, the size distribution of the virus was analyzed by DLS. The results of intensity distribution are shown. The red box indicates the particle size at the highest intensity

Finally, we examined whether GTE treatment affects the morphological properties of the influenza virus. The PR8 virus was treated with 0.1% GTE or PBS and the virus particles were observed under cryo-electron microscope (cryo-EM). As shown Fig. 5a, the treatment of GTE did not result in noticeable changes in particle sizes and the shape of the viruses, as compared with PBS control (Fig. 5). Furthermore, the size distribution of the virus particles was measured using dynamic light scattering (DLS) system. PR8 virus treated with PBS showed the highest intensity at particle diameter of 97.8 nm (Fig. 5b). GTE treatment resulted in the highest intensity at particle diameter of 126.2 nm, 113.7 nm, and 121 nm for GTE concentration of 0.01%, 0.05%, and 0.1%, respectively, showing that GTE treatment increased the viral particle sizes to 15.5 − 28.8%. Considering that the influenza virus particles typically have a size of 80 − 120 nm [36], the increase in the particle sizes by GTE treatment does not appears to be significant. Catechins may also interact with the viral membranes as well as the viral proteins [22]. Whether the observed changes in the particle size may be associated with the membrane fluidity/integrity, crucially important for viral membrane fusion for infection, remains to be further investigated.

## Discussion

The primary goal of the present study was to evaluate GTE as potential virucidal disinfectant. We examined the temporal stability of virucidal activity of GTE against the human influenza A/H1N1 virus and the effects of antioxidant supplementation on its stability. It has been shown that green tea catechines or green tea extract exert potent antiviral effects against a variety of human infecting viruses including influenza viruses [32, 37]. Previous studies have mainly focused on the targets and inhibitory actions of green tea catechins at various stages of viral replication cycles [22, 38, 39]. The present data showed that the virucidal effects of GTE can be maintained up to eight weeks in a solution type and for 16 weeks in a powder type, respectively, for the entire duration of experiments conducted. A long-term maintenance of virucidal activity could be related to the chemical stability of catechins [40]. The concentrations of green tea catechins were measured over time and there were no significant differences in the concentrations between GTE and GTE-mix solutions (Fig. 4a). The data showed that the catechin concentrations remained constant over the tested period, implying that the additive antioxidant may not directly influence the chemical stability of catechins. The inactivating activities of 0.05% and 0.01% GTE steadily decreased as storage time increased (Fig. 2). Given that the concentration of catechins remained constant over the prolonged storage as shown in Fig. 4a, it is likely that crosslinking efficiency of the catechins to the viral proteins/membranes was decreased during the storage, for which the precise molecular mechanism remains to be elucidated further delicate studies.

The potential effect of antioxidants was also evaluated, in the form of mixture (GTE-mix) comprising ascorbic acid, citric acid, and sodium benzoate (common food preservatives). It is well documented that ascorbic acid is one of the strongest reductants and acts as an oxygen scavenger [41, 42]. Although our experimental condition using the ascorbic acid did not perfectly reflect anaerobic condition, our data clearly showed that the ascorbic acid delayed the GTE-mediated viral inactivation, most likely through inhibiting the autoxidation of catechins mediated by molecular oxygen. The data suggest that the potency of virucidal activity was compromised for the initial period of a week; the reduction of infectious viral titer for GTE and GTE-mix was 4–6 log_10_ and 1–3 log_10_, respectively. However, the virucidal activity of GTE-mix persisted up to 8 weeks (Fig. 3a, b), in clear contrast to GTE without additives where the virucidal activity become exhausted after two weeks (Fig. 2). Thus, the combination of GTE with ascorbic acid as antioxidant resulted in a sustained virucidal activity for extended period. It should be noted that contrasting activities, pro-oxidant and anti-oxidant, have been documented with green tea catechins [35]. Moreover, it has been proposed that the catechins in green tea crosslink with cysteine residues in a protein through autoxidation process [43, 44]. Thus, it is likely that a strong antioxidant such as ascorbic acid prevents the oxidation of polyphenolic OH groups of the catechins into quinone, a prerequisite for oxidative crosslinking to viral proteins [45]. Our data show that the viral inactivating activity of GTE-mix recovered after 14 days of storage despite the presence of ascorbic acid and other supplements (Fig. 3a, b). Investigation of the chemical or biological status of ascorbic acid as an antioxidant especially in the mixture with GTE during the storage could provide a possible explanation for this phenomenon. Intriguingly, the antiviral activities of 0.1% GTE-mix and those of lower concentrations of GTE-mix were differently affected by storage temperatures, as compared to those of GTE. After storage at 25 °C, the antiviral activity of 0.1% GTE-mix was reduced mildly, whereas those of 0.05% and 0.01% GTE-mix were considerably dampened (Fig. 3a). Conversely, after storage at 37 °C, the reduction of antiviral activity was more pronounced in 0.1% GTE-mix than those in 0.05% and 0.01% GTE-mix (Fig. 3b). Although, the addition of the supplements including antioxidant obviously influenced the changes in the antiviral kinetics, further detailed analyses would be required to address the issue. A recent report suggested that urea supplementation of alcohol-based disinfectants in the presence of citric acid may increase their antiviral effects against a number of viruses [46]. Whether supplementing GTE with such components may enhance the stability of antiviral effect of GTE remains to be further tested.

With promising outcome of long-term stability of virucidal effects of GTE, it is likely that potent antiviral activity could be extended to other viruses, only to underscore a general and broader use of GTE as an effective disinfectant against viral infections. The antiviral spectrum of GTE includes not only enveloped viruses such as influenza virus but also non-enveloped viruses such as parvovirus as well [47]. The present results could be usefully implemented for the development of the first-hand personal and public hygiene for lessening the medical burden associated with emerging and re-emerging viral infections.

## Conclusions

The durability of antiviral effect of the GTE against human influenza virus (H1N1) was examined as a powder type and a solution type over extended periods at various temperature conditions. The data revealed that a potent antiviral activity of the GTE with about 4 log_10_ reduction of viral titers was maintained with wide range of temperatures (4–37 °C) for two months as solution type, and for prolonged period as power. These data suggest that catechin-based antiviral agents could be formulated as a safe and environmentally friendly personal hygiene against viral infections. When formulated with ascorbic acid, the potency of virucidal activity was temporarily compromised, while the exhaustion of antiviral activity was delayed, prolonging the duration of the virucidal function over extended period. In contrasts to most antiviral agents of synthetic chemicals, GTE does not pose any safety concern, but even considered beneficial for human health. Besides human use, it could also be used for protecting animals and livestock from viral infections and reducing zoonotic transmissions. The GTE could be supplemented with other components for an extended use as environmentally friendly virucidal agents.

## Abbreviations

Cryo-EM: Cryo-electron microscopy
DLS: Dynamic light scattering
EC: Epicatechin
ECG: Epicatechin-gallate
EGC: Epigallocatechin
EGCG: Epigallocatechin-3-gallate
FID: Flame ionization detector
GTE: Green tea extract
GTE-mix: GTE solution supplemented with 2% citric acid, 0.1% sodium benzoate, and 0.2% ascorbic acid
PFU: Plaque forming unit
